# Hierarchical structural component model for pathway analysis of common variants

**DOI:** 10.1186/s12920-019-0650-0

**Published:** 2020-02-24

**Authors:** Nan Jiang, Sungyoung Lee, Taesung Park

**Affiliations:** 1 Interdisciplinary Program in Bioinformatics, Seoul National University, Seoul, 08826 Korea; 2 Center for Precision Medicine, Seoul National University Hospital, Seoul, 03080 Korea; 3 Department of Statistics, Seoul National University, Seoul, 08826 Korea

**Keywords:** Common variants, Genome-wide association study, Hierarchical components, Pathway analysis

## Abstract

**Background:**

Genome-wide association studies (GWAS) have been widely used to identify phenotype-related genetic variants using many statistical methods, such as logistic and linear regression. However, GWAS-identified SNPs, as identified with stringent statistical significance, explain just a small portion of the overall estimated genetic heritability. To address this ‘missing heritability’ issue, gene- and pathway-based analysis, and biological mechanisms, have been used for many GWAS studies. However, many of these methods often neglect the correlation between genes and between pathways.

**Methods:**

We constructed a hierarchical component model that considers correlations both between genes and between pathways. Based on this model, we propose a novel pathway analysis method for GWAS datasets, Hierarchical structural Component Model for Pathway analysis of Common vAriants (HisCoM-PCA). HisCoM-PCA first summarizes the common variants of each gene, first at the gene-level, and then analyzes all pathways simultaneously by ridge-type penalization of both the gene and pathway effects on the phenotype. Statistical significance of the gene and pathway coefficients can be examined by permutation tests.

**Results:**

Using the simulation data set of Genetic Analysis Workshop 17 (GAW17), for both binary and continuous phenotypes, we showed that HisCoM-PCA well-controlled type I error, and had a higher empirical power compared to several other methods. In addition, we applied our method to a SNP chip dataset of KARE for four human physiologic traits: (1) type 2 diabetes; (2) hypertension; (3) systolic blood pressure; and (4) diastolic blood pressure. Those results showed that HisCoM-PCA could successfully identify signal pathways with superior statistical and biological significance.

**Conclusions:**

Our approach has the advantage of providing an intuitive biological interpretation for associations between common variants and phenotypes, via pathway information, potentially addressing the missing heritability conundrum.

## Background

Genome-wide association studies (GWAS) have greatly advanced our understanding of the association between sets of genetic variants (genotypes) and traits of interest (phenotypes). GWAS typically focus on associations between single-nucleotide polymorphisms (SNPs) and traits (phenotypes), such as type 2 diabetes (T2D) [1]. To identify common variants in GWAS, many statistical methods, including logistic and linear regression, have been widely used. Since most of these methods are based on single variant analysis, their statistically significant results sometimes may suffer from a lack of biological interpretation. In addition, it has been reported that only a small portion of the total heritability, of specific traits, can be explained by these identified SNPs [2]. To enhance interpretation of SNP association results, many gene-based and pathway-based association analysis methods have been developed. Biological pathways, which complexly interact with each other, always have more direct influence on related biological behaviors, as compared to genes [3]. Thus, it is easier to interpret pathway-based results than SNP-based results. Such pathway-based association methods, developed for GWAS, often identify pathways based on results from a single analysis of SNPs. These methods often use only the most statistically significant SNPs, according to the *p*-values obtained from single SNP analysis. However, such analyses ignore genetic information from the SNPs that are not selected [4–6]. In addition, high correlations always exist between pathways, potentially arising from many genes shared between pathways. Thus, methods neglecting these correlations may mislead phenotype association results [7].

Considering these deficiencies, a hierarchical component model has been constructed, PHARAOH (Pathway-based approach using HierArchical components of collapsed RAre variants Of High-throughput sequencing data). PHARAOH performs pathway analysis for rare variants using a single hierarchical model, and includes a collapsing step for rare variants, whose data are usually sparse. PHARAOH gene-level summary statistics are obtained by a special weight approach for rare variants, and analyzes entire genes and pathways by adding ridge-type penalties on both gene and pathway effects on traits [8]. PHARAOH is usually used to analyze rare variants, rather than common variants, due to the special collapsing step, since common variant data usually needs dimension reduction instead of collapsing. In this study, we utilized the main framework of PHARAOH, and principal component analysis (PCA), to construct a hierarchical component model for common variants. Based on this model, we proposed a novel pathway analysis method for GWAS datasets, named Hierarchical structural Component Model for Pathway analysis of Common vAriants (HisCoM-PCA).

HisCoM-PCA has several distinctive features. First, HisCoM-PCA can identify associations between a distinct trait and entire pathways, using a single model. It can simultaneously quantify both the effects of pathways and genes to the phenotype. Second, HisCoM-PCA performs pathway analysis using gene-level summary statistics from SNPs within the same genes. Third, HisCoM-PCA allows potential correlations between genes and between pathways by adding ridge-type penalties to both genes and pathways effects. In addition, HisCoM-PCA may not only be used for binary phenotypes, but also continuous phenotypes. Overall, HisCoM-PCA can identify associated genes and pathways, by controlling correlations within them.

In this study, we applied HisCoM-PCA for two binary phenotypes, type 2 diabetes (T2D) and hypertension (HT), and two continuous phenotypes, systolic blood pressure (SBP) and diastolic blood pressure (DBP), using large-scale SNP data from a Korean population study, KARE (8840 samples) [9], and the KEGG pathway database (186 pathways) [10]. Furthermore, HisCoM-PCA was compared to three existing pathway-based approaches: GSA-SNP2 [4], sARTP [11], and MAGMA [12]. To check the power and type I error of HisCoM-PCA, a simulation study was performed using the Genetic Analysis Workshop (GAW) 17 generated dataset [13]. The empirical power of HisCoM-PCA was then compared to three other existing methods. The results of both a simulation study and real data analysis demonstrated that HisCoM-PCA could successfully identify statistically associated and biologically plausible pathways, for complex traits of interest.

## Methods

### KARE cohort dataset

The Korea Association REsource (KARE project) is a nearly 9000-participant cohort GWAS study of Korean populations from Ansan and Ansung, representing city and countryside populations, respectively [9]. The common variant genotype data of 8840 individuals were generated using the Affymetrix Genome-Wide Human SNP array 5.0. This chip consists of about 50 million autosomal SNPs, with a total of 352,228 SNPs available after quality control. In this study, we excluded SNPs with minor allele frequencies (MAFs) ≤ 0.05, genotype calling rates < 95%, and Hardy-Weinberg equilibrium *p*-values < 10^−6^. Thus, we only kept the subjects with gender consistencies, and those whose calling rates were > 90%. After such quality control processes, missing values were imputed only for existing variants.

### Definition of type 2 diabetes

An individual is defined as T2D, according to the following criteria: (1) under treatment for T2D; (2) fasting plasma glucose (FPG) ≥ 126 mg/dL, 2-h postprandial blood glucose (Glu120) ≥ 200 mg/dL, or glycated hemoglobin (HbA1c) ≥ 6.5%; and (3) age of disease onset ≥40 years. Resultantly, a total of 1288 subjects were diagnosed as T2D, among 8840 individuals, with another 3687 individuals selected as normal subjects by the inclusion criteria: (1) FPG < 100 mg/dL, Glu120 < 140 mg/dL and HbA1c < 5.7%; and (2) no history of diabetes [14]. Demographic variables of the 4974 selected subjects are summarized in Table 1.

**Table 1:** Demographic variables for KARE cohort (T2D)

	T2D subjects	Normal subjects
Area (Ansan/Ansung)	673/615	1607/2080
Gender (Male/Female)	671/617	1679/2008
Age (Mean ± SD)	55.92(± 8.80)	49.88(± 8.31)
BMI (Mean ± SD)	25.54(± 3.27)	24.10(± 2.90)
Number of subjects	1288	3687

*SD* standard deviation, *BMI* body mass index

### Definition of hypertension

A total of 2008 individuals were defined as hypertensive, according to the following criteria: (1) SBP ≥ 140 mmHg and/or DBP ≥ 90 mmHg; and (2) treatment with antihypertension medication, while 4569 individuals were defined as normotensive controls according to the criteria: SBP < 120 mmHg and DBP < 80 mmHg. Subjects with pre-hypertensive status were excluded from the analysis. For quantitative trait analysis of SBP and DBP, 1019 subjects were excluded due to hypertensive therapy or drug treatments, variables that influence blood pressure [15]. The basic characteristics and blood pressure of the subjects are listed in Table 2.

**Table 2:** Basic characteristics of study subjects

(a) Basic characteristics of hypertensive cases and normotensive controls		
	HT subjects	Normal subjects
Area(Ansan/Ansung)	1204/804	1756/2813
Gender(Male/Female)	916/1092	2065/2504
Age(Mean ±SD)	56.74(± 8.42)	49.43(± 8.09)
BMI(Mean ±SD)	25.62(± 3.27)	24.03(± 2.94)
Number of subjects	2008	4569
(b) Basic characteristics of subjects for blood pressure analysis		
	Subjects	
Area(Ansan/Ansung)	3591/4225	
Gender(Male/Female)	3784/4032	
Age(Mean ±SD)	51.45(± 8.74)	
BMI(Mean ±SD)	24.40(± 3.07)	
SBP(Mean ±SD)	115.56(± 17.22)	
DBP(Mean ±SD)	74.11(± 11.24)	
Number of subjects	7816	

*SD* standard deviation, *BMI* body mass index, *SBP* systolic blood pressure, *DBP* diastolic blood pressure

### HisCoM-PCA

#### Step 1: SNPs dimension reduction by principal component analysis (PCA)

The first step of HisCoM-PCA reduces the dimensions of the common variants, located in the same genes, by PCA. After PCA was performed for each gene, part of principle components (PCs), as gene-level summary statistics, are chosen to represent the corresponding genes. In order to reduce the high dimension of SNP data, we only select a small number of PCs for each gene for the next pathway analysis. In order to determine the number of PCs for used for the pathway analysis, we use the following simple criteria: (1) using only the first PC and (2) using the PCs whose cumulative proportion of variances are more than 30%. We use a R’s function prcomp in the stats package to conduct PCA.

#### Step 2: pathway analysis with a hierarchical component model (HisCoM)

After reducing the dimensions of common variants for each gene, pathway analysis is performed, using the selected PCs, with a hierarchical component model, as previously used for pathway analysis of rare variants [8]. Before the analysis, genes are mapped to the well-defined pathways in the pathway databases such as the Kyoto Encyclopedia of Genes and Genome (KEGG). Then, the PCs for these mapped genes are derived and assigned to the corresponding pathways. These PCs are used as input dataset for step 2.

**Fig. 1:**
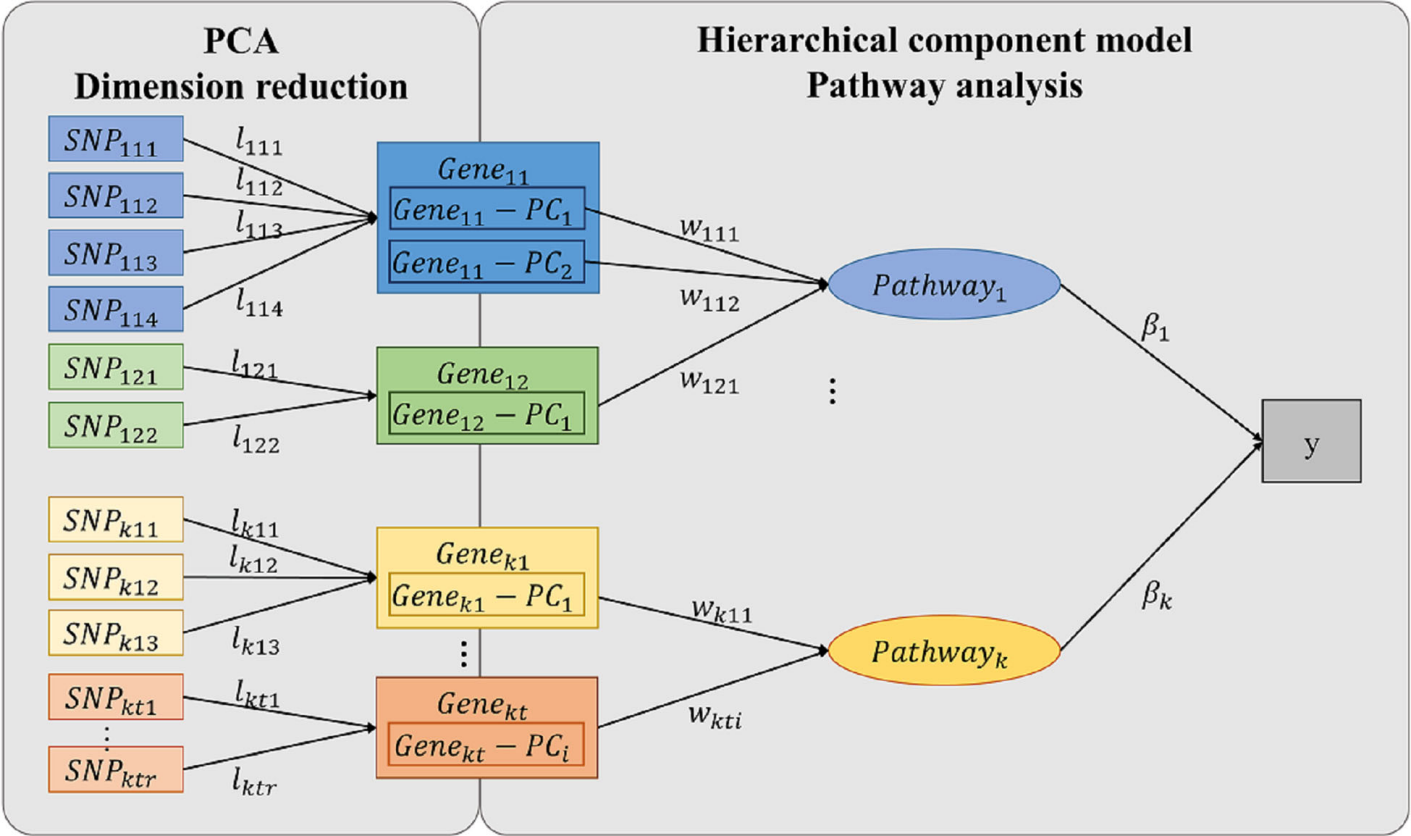
A schematic diagram of HisCoM-PCA *y* is the phenotype of subject; *pathway*_*k*_ means *k*^*th*^ pathway; *Gene*_*kt*_ − *PC*_*i*_ denotes *i*^*th*^ PC of the *t*^*th*^ gene in *k*^*th*^ pathway; *SNP*_*ktr*_ is *r*^*th*^ SNP of the *t*^*th*^ gene in *k*^*th*^ pathway; *l*_*ktr*_ denotes a loading value of *SNP*_*ktr*_ from PCA; *w*_*kti*_ denotes the weight assigned to *Gene*_*kt*_ − *PC*_*i*_; *β*_*k*_ denote the coefficient connecting the *k*^*th*^ pathway to the phenotype

In this model, pathways are defined as a weighted component of a set of PCs (Fig. 1). Let us define *y*_*j*_ as the phenotype of the *j*^*th*^ subject and assume that phenotype independently follow an exponential family distribution (*j* = 1, …, *N*). Let *K* be the number of pathways, *T*_*k*_ be the number of genes in the *k*^*th*^ pathway and *N*_*kt*_ be the number of PCs for the *t*^*th*^ gene in the *k*^*th*^ pathway. Let *g*_*kti*_ denote the *i*^*th*^ PC derived from PCA at the step 1 (*k* = 1, …, *K*; *t* = 1, …, *T*_*k*_; *i* = 1, …, *N*_*kt*_). These *g*_*kti*_ s represent the genes and have continuous values. Let *w*_*kti*_ denote a weight assigned to *g*_*kti*_ and *β*_*k*_ denote the coefficient connecting the *k*^*th*^ pathway to the phenotype. For each individual, the relationships between PCs and binary phenotype are established in such a way that:

$$ logit\left(\pi \right)={\beta}_0+\sum \limits_{k=1}^K\left[\sum \limits_{t=1}^{T_k}\sum \limits_{i=1}^{N_{kt}}{g}_{kt i}{w}_{kt i}\right]{\beta}_k $$

To estimate the parameters in HisCoM-PCA, we use the alternating least squares (ALS) algorithm. The ALS algorithm was originally proposed by de Leeuw et al. [16] and adopted by Hwang and Takane for the generalized structural component analysis (GSCA) [17], and later by Lee et al. for the penalized log-likelihood function [8]. The ALS algorithm minimizes the objective function in the framework of least squares estimation. We use the ALS algorithm for the penalized log-likelihood function of Lee et al. [8]. Our ALS algorithm consists of two steps and these two steps iterate until convergence.

*Step*2 − 1: For fixing the weight coefficient estimates *w*_*kti*_, update the pathway coefficient estimates *β*_*k*_, in the sense of least squares.

*Step*2 − 2: For fixing pathway coefficient estimates *β*_*k*_, update the weight coefficient estimates *w*_*kti*_, in the sense of least squares.

To take into account potential correlations between genes and between pathways, we utilize a penalization approach. In this study, we adopt a ridge-type penalty to control multi-collinearity between genes and between pathways. Then, we sought to maximize the penalized log-likelihood function, given as follows:

$$ \phi ={\sum}_{j=1}^N\log p\left({y}_j;{\gamma}_j,\delta \right)-\frac{1}{2}{\lambda}_g{\sum}_{k=1}^K{\sum}_{t=1}^{T_k}{\sum}_{i=1}^{N_{kt}}{w}_{kt i}^2-\frac{1}{2}{\lambda}_p{\sum}_{k=1}^K{\beta}_k^2, $$

where *p*(*y*_*j*_; *γ*_j_, *δ*) is the probability distribution for the phenotype of the *j*^*th*^ individual, and *λ*_*g*_ and *λ*_*p*_ are ridge parameters for genes and pathways, respectively. After estimation, we performed permutation testing by resampling the phenotypes, to test the significance of the parameters. Here, we use a tool called WISARD (Workbench for Integrated Superfast Association study with Related Data) [18], which was developed for fast and a comprehensive analysis of SNP-chip and next-generation sequencing data. WISARD can perform the standard pathway analysis with SNP data as input. Instead of SNPs, we use the PCs derived from step 1 as input of WISARD to perform our PC based pathway analysis.

### Simulation study

To check the power and type Ι error rate of HisCoM-PCA, a simulation study was performed using simulation data from the Genetic Analysis Workshop 17 (GAW17) [13]. In brief, a GAW17 simulation dataset was generated for 697 individuals from the 1000 Genomes Project [19], containing 24,487 SNVs and four phenotypes (Q1, Q2, Q4, and AFFECTED). The SNPs with minor allele frequencies (MAFs) ≤ 0.05, or genotype calling rates < 95% were excluded in the simulation study. We also kept the subjects with gender consistencies, and those whose calling rates were > 90%. Among the four simulated phenotypes, only Q1 was generated using pathway information, and was simulated to be affected by 9 genes from the vascular endothelial growth factor (VEGF) pathway, as defined by Ingenuity Pathway Analysis [20]. We next examined the power according to the proportion of identifying the VEGF pathway from the entirety of pathways in the KEGG database. Type I error of HisCoM-PCA was examined by the proportion of identifying null pathways which did not contain causal genes. Both type I error and power were calculated by analysis for Q1. To compare the power with other existing methods, we also analyzed the GAW17 dataset using sARTP [11], a self-contained version of MAGMA, a competitive version of MAGMA [12] and GSA-SNP2 [4].

## Results

### Simulation study using the gene analysis workshop 17 (GAW17) dataset

To check the power and type I error of HisCoM-PCA, we performed a simulation study using the GAW17 dataset, for both binary and continuous types of a Q1 trait. For binary phenotypes, we transformed the continuous values of Q1 to binary values, using the median. Each SNP was then assigned to a gene, if its location was in, or within 20 kb of, the gene, and the KEGG database then used to map genes and pathways. In the simulation study, we chose the first PCs and PCs whose cumulative proportion of variances was more than 30%, after PCA of each gene. The tuning parameters of our method, *λ*_*g*_ and *λ*_*p*_, were optimized based on five-fold CV.

**Fig. 2:**
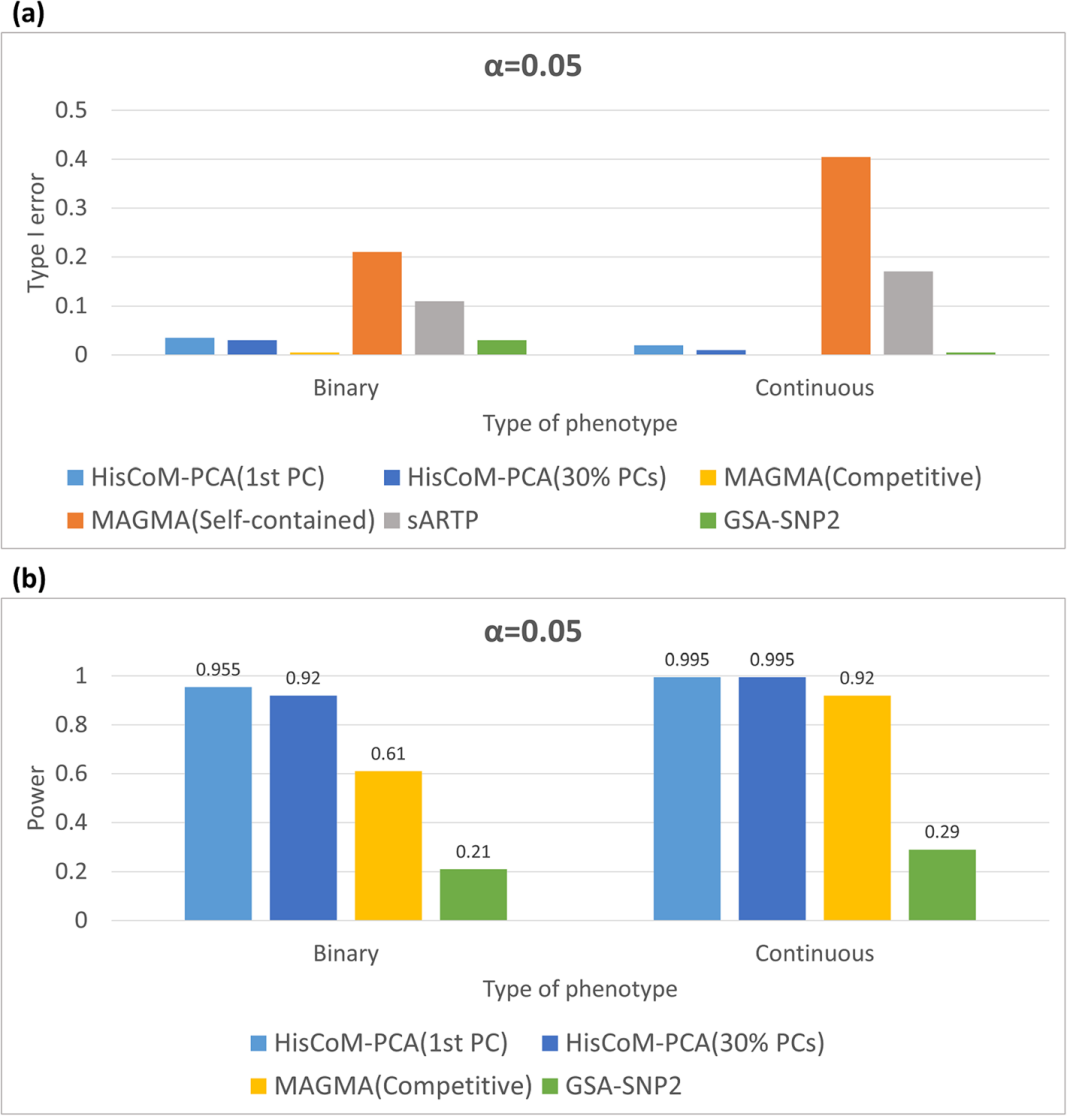
Empirical type I errors and powers of HisCoM-PCA and other methods (**a**) Empirical type I errors of HisCoM-PCA, sARTP, two versions of MAGMA, and GSA-SNP2. Empirical type I error indicates the times of identifying a null pathway among 200 replicates. (**b**) Empirical powers of HisCoM-PCA, competitive version of MAGMA and GSA-SNP2. Empirical power indicates the times of identifying VEGF pathway among 200 replicates

To investigate where the type I error rate is controlled, we examined type I error by the proportion of identifying a null pathway whose number of genes was the same as the VEGF pathway. We checked the type I errors of HisCoM-PCA, sARTP, competitive version of MAGMA, self-contained version of MAGMA, and GSA-SNP2 (Fig. 2).

To that end, HisCoM-PCA controlled type I error with PC selection criteria. GSA-SNP2, and the competitive version of MAGMA, also controlled type I error well. However, the type I errors of sARTP, and the self-contained version of MAGMA, were too inflated. Thus, we only compared the power of HisCoM-PCA with GSA-SNP2, and the competitive version of MAGMA. To examine the power, we calculated proportion of identifying VEGF pathways from 168 KEGG pathways, with 200 replicates. The powers of the three methods are shown in Fig. 2.

For both continuous and binary phenotypes, HisCoM-PCA showed the highest power, compared to the other methods. The powers of HisCoM-PCA, with two types of phenotypes, and two criteria of PC selection, were all higher than 0.95. However, the power of GSA-SNP2 were only 0.21 for the binary phenotype and 0.29 for the continuous phenotype, respectively. While MAGMA showed higher power than GSA-SNP2, it showed only 0.6 power for the binary phenotype. However, HisCoM-PCA showed similar powers with either PC selection criteria, while all the methods showed higher power with continuous vs. binary phenotypes.

### Real data analysis of common variants from KARE

For KARE data, PLINK 1.90 [21] was used to perform quality control analysis using the criteria described in the Materials section. The SNPs were mapped to the UCSC hg19 genomic coordination. Missing genotype data was imputed using the Beagle 5.0 [22] software program. Then, the SNPs were annotated with genes using SnpEff v.4.3 [23]. After mapping these genes to the KEGG pathway database, a total of 3996 genes were matched to 186 KEGG pathways. The distribution of the number of SNPs per gene is given in Additional file 1: Figure S1. For the 3996 genes, we used the following simple criteria to choose the number of PCs: (1) using only the first PC and (2) using the PCs whose cumulative proportion of variances were more than 30%. When using the first criterion, each gene used only one PC. Thus, there were 3996 PCs. On the other hand, when using the second criterion, one gene could have multiple PCs. As a result, 4486 PCs were used. We then performed pathway analysis for four phenotypes: type-2 diabetes (T2D), hypertension (HT), systolic blood pressure (SBP), and diastolic blood pressure (DBP). Following association tests conducted in other previous studies of the KARE dataset, age, sex, body mass index (BMI), and area were included as covariates in the pathway analysis. In addition to HisCoM-PCA, other existing methods such as sARTP, MAGMA (self-contained and competitive versions) and GSA-SNP2 were used for comparison. The tuning parameters *λ*_*g*_ and *λ*_*p*_, were chosen based on five-fold cross-validation (CV). To test the pathways’ significance, we performed permutation tests by generating 1000 permuted phenotypes.

HisCoM-PCA, using the first PCs, successfully identified 14 pathways for T2D, 15 pathways for HT, 3 pathways for SBP, and 9 pathways for DBP, respectively, at a 5% significance level. HisCoM-PCA, using the 4486 PCs, identified 13 pathways for T2D, 20 pathways for HT, 6 pathways for SBP, and 7 pathways for DBP, respectively, at the same significance level. These different PC selection criteria provided very consistent results. Both identified 10 common pathways for T2D, 14 common pathways for HT, three common pathways for SBP, and five common pathways for DBP. The summary results of GSA-SNP2, MAGMA (competitive version) and HisCoM-PCA for KARE data analysis are shown in Additional file 1: Figure S2. As a multiple testing correction method, the false discovery rate (FDR) was used to calculate corrected *p*-values for each pathway. When HisCoM-PCA was used for the first PCs, only three pathways had FDR corrected p-values less than 0.1 for HT. However, none of pathways passed this threshold for other phenotypes. When HisCoM-PCA was used for the PCs whose cumulative proportion of variance is more than 30%, two pathways for T2D and one pathway for HT passed the same threshold of FDR corrected p-value, respectively. None of pathways passed this threshold for SBP and DBP.

For T2D analysis, HisCoM-PCA successfully identified several well-known pathways biologically related to T2D. For example, pathways such as calcium signaling, the renin-angiotensin system, and phosphatidylinositol signaling, are known to be related to insulin resistance or insulin sensitivity [24–27]. Of these, calcium signaling is crucial for insulin secretion in pancreatic β-cells [24, 25], while phosphatidylinositol signaling is known to play an important role in an insulin-stimulated glucose metabolism pathway associated with obesity and T2D [27]. Moreover, some diseases, such as Alzheimer’s disease (AD), asthma, and dilated cardiomyopathy have been reported to share molecular pathways or risk factors with T2D [28–31], and several studies have shown that insulin resistance is related to risk of AD, as well as T2D [28]. These results demonstratee that application of HisCoM-PCA to T2D successfully identified various pathways of these diseases. In addition, folate biosynthesis and hedgehog signaling have also been reported to potentially relate to T2D [32, 33]. These pathway results for T2D, using HisCoM-PCA, and the other four methods, are summarized in Table 3.

**Table 3:** Pathways identified for T2D

Pathway	HisCoM-PCA^a^	HisCoM-PCA^b^	MAGMA	GSA-SNP2
	*P* value (q-value)			
folate biosynthesis	0.004 (0.1518)	0.002 (0.0633)	0.0537 (0.891)	0.0019 (0.2936)
hedgehog signaling pathway	0.006 (0.1627)	0.016 (0.2531)	0.3073 (0.917)	0.028 (0.5284)
olfactory transduction^*^	0.006 (0.1627)	0.002 (0.0633)	0.0482 (0.891)	0.0036 (0.2936)
biosynthesis of unsaturated fatty acids	0.01 (0.2373)	0.012 (0.2071)	0.089 (0.891)	0.0373 (0.5284)
Alzheimer’s disease	0.014 (0.2657)	0.036 (0.4555)	0.1808 (0.891)	0.0446 (0.5284)
calcium signaling pathway	0.014 (0.2657)	0.026 (0.3796)	0.0791 0.891)	0.0329 (0.5284)
asthma	0.016 (0.2761)	0.008 (0.1687)	0.0517 0.891)	0.0737 (0.6529)
acute myeloid leukemia	0.032 (0.4302)	0.042 (0.4621)	0.0781 (0.891)	0.5442 (1)
melanogenesis	0.034 (0.4302)	0.012 (0.2071)	0.0786 (0.891)	0.0223 (0.5284)
long term potentiation	0.04 (0.4466)	0.028 (0.3796)	0.1284 (0.891)	0.1616 (0.8349)
phosphatidylinositol signaling system	0.1119 (0.5185)	0.006 (0.1424)	0.7656 (0.9778)	0.1107 (0.7355)
dilated cardiomyopathy	0.03 (0.4302)	0.0819 (0.5188)	0.3994 (0.917)	0.0408 (0.5284)
renin angiotensin system	0.038 (0.4466)	0.0539 (0.4621)	0.3794 (0.917)	0.0917 (0.6923)

HisCoM-PCA^a^ is HisCoM-PCA with the first PC of each gene. HisCoM-PCA^b^ is HisCoM-PCA with the PCs whose cumulative proportion of variance is more than 30%. MAGMA is competitive version of MAGMA. The q-value is the FDR corrected p-value. Pathway with “*” was identified by 3 methods

The pathways related to blood pressure (BP) were also identified by HisCoM-PCA using the phenotypes HT, SBP, and DBP. In that regard, calcium signaling pathway and the complement and coagulation cascades pathway were previously shown to be related to BP regulation [34, 35]. BP regulation is influenced by regulators of vascular tone, which are dependent on ion channels, such as voltage-gated Ca^2+^ channels, members of the calcium signaling pathway. Moreover, the kallikrein-kinin system (KKS) importantly regulates BP by influencing vascular tone and renal salt processing. It is also well known that KKS is a large picture of the complement and coagulation cascades pathway. Other disease pathways, such as maturity onset diabetes of the young (MODY) and hypertrophic cardiomyopathy (HCM) were identified by HisCoM-PCA, and previous studies have shown that MODY and HCM may also associate with HP [36–38]. These pathway results for HT, SBP, and DBP, using HisCoM-PCA and the other four methods, are shown in Table 4.

**Table 4:** Pathways identified for BP

Pathway	HisCoM-PCA^a^	HisCoM-PCA^b^	MAGMA	GSA-SNP2
	P value (q-value)			
inositol phosphate metabolism	0.002 (0.0633)	0.004 (0.1265)	0.1966 (1)	9.008e-06 (0.0017)
phosphatidylinositol signaling system	0.002(0.0633)	0.004 (0.1265)	0.2256 (1)	1e-04 (0.013)
ubiquitin mediated proteolysis	0.002 (0.0633)	0.002 (0.0949)	0.1076 (1)	0.5963 (0.9839)
calcium signaling pathway	0.008 (0.2169)	0.006 (0.1627)	0.0994 (1)	0.0228 (0.4708)
neurotrophin signaling pathway	0.01 (0.2373)	0.01 (0.2373)	0.6801 (1)	0.3157 (0.971)
epithelial cell signaling in helicobacter pylori infection	0.012 (0.2531)	0.05 (0.4126)	0.0826 (1)	0.1245 (0.7719)
complement and coagulation cascades	0.014 (0.2657)	0.03 (0.3417)	0.1428 (1)	0.3405 (0.9839)
maturity onset diabetes of the young	0.018 (0.3106)	0.036 (0.3417)	0.067 (1)	0.0239 (0.4708)
snare interactions in vesicular transport	0.022 (0.348)	0.012 (0.2531)	0.2071 (1)	0.0653 (0.5878)
hypertrophic cardiomyopathy (HCM) ^*^	0.1259 (0.5573)	0.03 (0.3417)	0.0074(1)	3e-04 (0.0157)

HisCoM-PCA^a^ is HisCoM-PCA with the first PC of each gene. HisCoM-PCA^b^ is HisCoM-PCA with the PCs whose cumulative proportion of variance is more than 30%. MAGMA is competitive version of MAGMA. The q-value is the FDR corrected *p*-value. Pathway with “*” was identified by 3 methods

## Discussion

HisCoM-PCA is a novel method for pathway analysis of GWAS data. By applying HisCoM-PCA to a large population study dataset (KARE), we identified several biologically associated pathways for type-2 diabetes (T2D) and blood pressure (BP). For BP, we used three phenotypes: hypertension (HT), systolic blood pressure (SBP), and diastolic blood pressure (DBP). Whether the phenotype of interest is continuous or binary, HisCoM-PCA can successfully detect associated pathways with statistical significance. As self-directed validation, some pathways related to HT were also identified for SBP or DBP, simultaneously providing significant *p*-values. Beside pathway analysis, we performed gene analysis using HisCoM-PCA at the same time. The reported genes identified by HisCoM-PCA at the 5% nominal significant level are summarized in Additional file 1: Tables S1 and S2. To that end, HisCoM-PCA identified several genes well known to genetically influence T2D or BP, demonstrating that HisCoM-PCA can detect both pathways and genes having biological significance.

Other existing pathway methods revealed numbers of significant pathways. As shown in simulation studies, however, they have high chance of being false positives. On the other hand, some pathways identified by HisCoM-PCA were previously reported to be related to T2D or BP, while these pathways were not significant by other pathway identification methods we used for comparison. In addition, some pathways were jointly identified by other methods and HisCoM-PCA. Real data analysis showed that HisCoM-PCA can provide new candidates that other methods cannot successfully identify.

We also examined empirical power and type I error rate for both binary and continuous phenotypes, using the Genetic Analysis Workshop 17 (GAW17) simulation dataset for GWAS. Compared to several methods, HisCoM-PCA controlled type I error well and showed high statistical power. However, some methods, such as sARTP and the self-contained version of MAGMA, did not control type I error well. Moreover, the methods that well controlled type I error showed lower power than HisCoM-PCA. In the simulation study, HisCoM-PCA analysis of the first PC showed similar power to HisCoM-PCA with PCs whose cumulative proportion of variance was more than 30%. This may indicate that the power is similar, using multiple PCs, which can save a lot of computing time.

## Conclusions

In this study, we proposed a novel pathway analysis method HisCoM-PCA for GWAS datasets. Over the existing methods, HisCoM-PCA has several advantages. HisCoM-PCA performs gene-based and pathway-based analysis directly from raw data, while GSA-SNP2 and MAGMA use only summary measures such as *p*-values or test statistics of univariate analysis. These are gene-level summary measures and are used as inputs to perform pathway analysis. However, since the values of these summaries do not directly represent the raw genetic data, this issue probably leads to false discoveries. In HisCoM-PCA, we can obtain gene-level summary statistics, by PCA, for each gene. These statistics are a linear combination of SNPs from the raw data. Using these values, subsequent analysis for genes and pathways may decrease the possibility of false discoveries.

HisCoM-PCA also considers correlations between pathways, an aspect usually neglected by other methods. Correlation between pathways may influence the combined effect of pathways on traits, similar to when correlations exist between genes in a specific pathway. To allow correlation between genes and between pathways, HisCoM-PCA applies a ridge-type penalization approach on coefficient estimation for both genes and pathways by analyzing entire pathways simultaneously. Cross-validation is then used to detect the optimal tuning parameters of ridge-type penalties. Note that other methods can only analyze one pathway at a time. For the mapped 186 pathways in our KARE data, HisCoM-PCA analyzed them with one big model, while other methods performed single pathway analyses 186 times.

Furthermore, only HisCoM-PCA have an ability to perform conditional inference for identifying a novel pathway given known pathways. For example, for a given well-known pathway related to a trait of interest, HisCoM-PCA can only identify additional pathways for this given pathway. HisCoM-PCA can also perform stepwise selection of pathways by adding one pathway sequentially given the selected pathways.

In addition to the above advantages, HisCoM-PCA has high flexibility for users. First, PC selection criteria may be defined by the user. Second, users can perform both non-target and target pathway analysis. Since HisCoM-PCA controls the correlation between pathways, it is useful to detect associated pathways having similar molecular mechanisms. However, HisCoM-PCA has higher computational burden than other methods due to permutation test. We are currently working on developing an asymptotic test which can replace the permutation test. We strongly believe that our method, HisCoM-PCA, can be applied to any number of GWAS studies, resulting in the successful identification of genes and pathways associated with specific phenotypes.

## Supplementary information

**Additional file 1: Table S1.** Reported genes identified by HisCoM-PCA (the first PC) for type 2 diabetes. **Table S2.** Reported genes identified by HisCoM-PCA (the first PC) for blood pressure. **Figure S1.** Summary for number of SNPs in each gene excluding genes with one SNP. **Figure S2.** Summary of significant results from GSA-SNP2, MAGMA and HisCoM-PCA for four phenotypes: T2D, HT, SBP and DBP.

## Data Availability

The genotype data of KARE samples are available by sending a request to the Distribution desk of Korea Biobank Network, National Institute of Health, Korea.

## Abbreviations

AD: Alzheimer’s disease
ALS: Alternating regulated least squares
BMI: Body mass index
BP: Blood pressure
CV: Cross-validation
DBP: Diastolic blood pressure
FPG: Fasting plasma glucose
GAW17: Genetic analysis workshop 17
Glu120: 2-h Postprandial blood glucose
HbA1c: Glycated hemoglobin
HCM: Hypertrophic cardiomyopathy
HisCoM: Hierarchical component model
HisCoM-PCA: Hierarchical structural component model for pathway analysis of common variants
HT: Hypertension
KARE: Korea Association REsource
KEGG: Kyoto Encyclopedia of Genes and Genome
KKS: Kallikrein-kinin system
MAFs: Minor allele frequencies
MODY: Maturity onset diabetes of the young
PCA: Principal component analysis
PCs: Principal components
PHARAOH: Pathway-based approach using hierarchical components of collapsed rare variants of high-throughput sequencing data
SBP: Systolic blood pressure
SD: Standard deviation
T2D: Type 2 diabetes
VEGF: Vascular endothelial growth factor
WISARD: Workbench for integrated superfast association study with related data
