# Supplementary material for: Hierarchical structural component model for pathway analysis of common variants
Source: BMC Med Genomics. 2020 Feb 24;13(Suppl 3):26. doi: 10.1186/s12920-019-0650-0 (PMC7038534; doi:10.1186/s12920-019-0650-0)
Supplement: Supplementary file 1 — Additional file 1: Table S1. Reported genes identified by HisCoM-PCA (the first PC) for type 2 diabetes. Table S2. Reported genes identified by HisCoM-PCA (the first PC) for blood pressure. Figure S1. Summary for number of SNPs in each gene excluding genes with one SNP. Figure S2. Summary of significant results from GSA-SNP2, MAGMA and HisCoM-PCA for four phenotypes: T2D, HT, SBP and DBP. [file 12920_2019_650_MOESM1_ESM.docx]

**Table S1** Reported genes identified by HisCoM-PCA (the first PC) for type 2 diabetes

| Pathway | Gene | P-values | Weight |
| --- | --- | --- | --- |
| adherens junction | TCF7L2 | 0.0220 | 0.242 |
| allograft rejection | TNF | 0.0360 | -0.2994 |
| alzheimers disease | CACNA1C | 0.0400 | 0.2405 |
| amyotrophic lateral sclerosis als | TNF | 0.0060 | 0.3422 |
| apoptosis | TNF | 0.0100 | 0.2438 |
| arrhythmogenic right ventricular cardiomyopathy arvc | CACNA1C | 0.0060 | 0.3189 |
| basal cell carcinoma | TCF7L2 | 0.0320 | -0.2492 |
| calcium signaling pathway | CACNA1C | 0.0080 | 0.2126 |
| cardiac muscle contraction | CACNA1C | 0.0060 | 0.3788 |
| cell cycle | CDKN2B | 0.0320 | 0.1960 |
| chronic myeloid leukemia | CDKN2A | 0.0420 | 0.1835 |
| colorectal cancer | TCF7L2 | 0.0400 | 0.2759 |
| cytokine cytokine receptor interaction | TNF | 0.0340 | -0.0996 |
| cytokine cytokine receptor interaction | TNFSF4 | 0.0460 | 0.1444 |
| dilated cardiomyopathy | CACNA1C | 0.0140 | 0.2936 |
| fc epsilon ri signaling pathway | TNF | 0.0460 | -0.2341 |
| gnrh signaling pathway | CACNA1C | 0.0040 | 0.3184 |
| graft versus host disease | TNF | 0.0100 | -0.3671 |
| hematopoietic cell lineage | TNF | 0.0440 | -0.2266 |
| leishmania infection | TNF | 0.0480 | -0.2502 |
| long term potentiation | CACNA1C | 0.0200 | 0.3576 |
| mapk signaling pathway | CACNA1C | 0.0360 | 0.1941 |
| neuroactive ligand receptor interaction | MTNR1B | 0.0120 | 0.1683 |
| nod like receptor signaling pathway | TNF | 0.0280 | -0.2739 |
| pathways in cancer | TCF7L2 | 0.0440 | -0.1121 |
| rig i like receptor signaling pathway | TNF | 0.0380 | 0.2594 |
| T cell receptor signaling pathway | TNF | 0.0260 | 0.2037 |
| toll like receptor signaling pathway | TNF | 0.0300 | -0.2134 |
| type I diabetes mellitus | TNF | 0.0260 | -0.2872 |
| type II diabetes mellitus | KCNJ11 | 0.0020 | 0.4123 |
| type II diabetes mellitus | CACNA1C | 0.0080 | 0.4210 |
| vascular smooth muscle contraction | CACNA1C | 0.0020 | 0.2809 |

**Table S2** Reported genes identified by HisCoM-PCA (the first PC) for blood pressure

(a) Reported genes identified by HisCoM-PCA (the first PC) for hypertension phenotype

| Pathway | Gene | P-values | Weight |
| --- | --- | --- | --- |
| adipocytokine signaling pathway | PPARA | 0.0040 | 0.3338 |
| aldosterone regulated sodium reabsorption | SCNN1B | 0.0040 | -0.4383 |
| alzheimers disease | CACNA1C | 0.0100 | -0.2367 |
| arrhythmogenic right ventricular cardiomyopathy arvc | CACNA1C | 0.0220 | 0.3016 |
| calcium signaling pathway | ATP2B1 | 0.0020 | 0.4320 |
| calcium signaling pathway | CACNA1C | 0.0040 | -0.1861 |
| calcium signaling pathway | ADRA1B | 0.0260 | -0.1524 |
| cardiac muscle contraction | CACNA1C | 0.0040 | 0.3402 |
| cell adhesion molecules cams | SELE | 0.0460 | 0.1310 |
| chemokine signaling pathway | CSK | 0.0020 | 0.4107 |
| dilated cardiomyopathy | CACNA1C | 0.0120 | 0.2918 |
| epithelial cell signaling in helicobacter pylori infection | CSK | 0.0020 | 0.5358 |
| gnrh signaling pathway | CACNA1C | 0.0100 | -0.2795 |
| hypertrophic cardiomyopathy hcm | CACNA1C | 0.0080 | 0.2835 |
| long term potentiation | CACNA1C | 0.0200 | -0.3789 |
| mapk signaling pathway | CACNA1C | 0.0020 | -0.1862 |
| neuroactive ligand receptor interaction | ADRA1B | 0.0340 | -0.1267 |
| neurotrophin signaling pathway | CSK | 0.0020 | 0.4300 |
| ppar signaling pathway | PPARA | 0.0300 | 0.3397 |
| regulation of actin cytoskeleton | CSK | 0.0020 | 0.3519 |
| regulation of autophagy | ULK3 | 0.0020 | 0.4058 |
| type II diabetes mellitus | CACNA1C | 0.0420 | -0.4009 |
| vascular smooth muscle contraction | CACNA1C | 0.0160 | -0.2623 |
| vascular smooth muscle contraction | ADRA1B | 0.0440 | -0.2068 |

(b) Reported genes identified by HisCoM-PCA (the first PC) for systolic blood pressure phenotype

| Pathway | Gene | P-values | Weight |
| --- | --- | --- | --- |
| aldosterone regulated sodium reabsorption | NEDD4L | 0.0420 | -0.4798 |
| alzheimers disease | BACE1 | 0.0060 | -0.3224 |
| calcium signaling pathway | ATP2B1 | 0.0020 | -0.4617 |
| calcium signaling pathway | ADRA1B | 0.0120 | 0.2547 |
| chemokine signaling pathway | CSK | 0.0020 | -0.3154 |
| endocytosis | NEDD4L | 0.0300 | 0.1660 |
| epithelial cell signaling in helicobacter pylori infection | CSK | 0.0020 | 0.5588 |
| neuroactive ligand receptor interaction | ADRA1B | 0.0080 | -0.1693 |
| neurotrophin signaling pathway | CSK | 0.0020 | 0.4443 |
| regulation of actin cytoskeleton | CSK | 0.0020 | -0.2913 |
| regulation of autophagy | ULK3 | 0.0020 | -0.0507 |
| steroid hormone biosynthesis | CYP11B1 | 0.0020 | 0.5275 |
| steroid hormone biosynthesis | CYP11B2 | 0.0100 | 0.4947 |
| type II diabetes mellitus | KCNJ11 | 0.0320 | 0.6657 |
| ubiquitin mediated proteolysis | NEDD4L | 0.0020 | 0.1730 |
| ubiquitin mediated proteolysis | UBE3C | 0.0020 | -0.1275 |
| vascular smooth muscle contraction | ADRA1B | 0.0140 | 0.3229 |

(c) Reported genes identified by HisCoM-PCA (the first PC) for diastolic blood pressure phenotype

| Pathway | Gene | P-values | Weight |
| --- | --- | --- | --- |
| adipocytokine signaling pathway | PPARA | 0.0380 | 0.3824 |
| alzheimers disease | BACE1 | 0.0040 | -0.4499 |
| calcium signaling pathway | ATP2B1 | 0.0040 | 0.4225 |
| calcium signaling pathway | GNA14 | 0.0120 | -0.3123 |
| calcium signaling pathway | ADRA1B | 0.0220 | -0.2061 |
| chemokine signaling pathway | CSK | 0.0080 | -0.2531 |
| epithelial cell signaling in helicobacter pylori infection | CSK | 0.0020 | 0.5208 |
| hypertrophic cardiomyopathy hcm | RYR2 | 0.0120 | 0.2203 |
| hypertrophic cardiomyopathy hcm | CACNA1C | 0.0160 | 0.1229 |
| hypertrophic cardiomyopathy hcm | CACNA1S | 0.0280 | -0.1372 |
| neuroactive ligand receptor interaction | ADRA1B | 0.0080 | -0.1374 |
| neurotrophin signaling pathway | CSK | 0.0020 | 0.4030 |
| ppar signaling pathway | PPARA | 0.0180 | -0.2871 |
| regulation of actin cytoskeleton | CSK | 0.0020 | 0.2587 |
| regulation of autophagy | ULK3 | 0.0200 | 0.0408 |
| renin angiotensin system | AGTR1 | 0.0340 | 0.4682 |
| renin angiotensin system | AGTR1 | 0.0340 | 0.4682 |
| steroid hormone biosynthesis | CYP11B1 | 0.0420 | 0.3861 |
| ubiquitin mediated proteolysis | NEDD4L | 0.0020 | 0.1250 |
| ubiquitin mediated proteolysis | UBE3C | 0.0020 | -0.1903 |
| vascular smooth muscle contraction | ADRA1B | 0.0280 | 0.2710 |


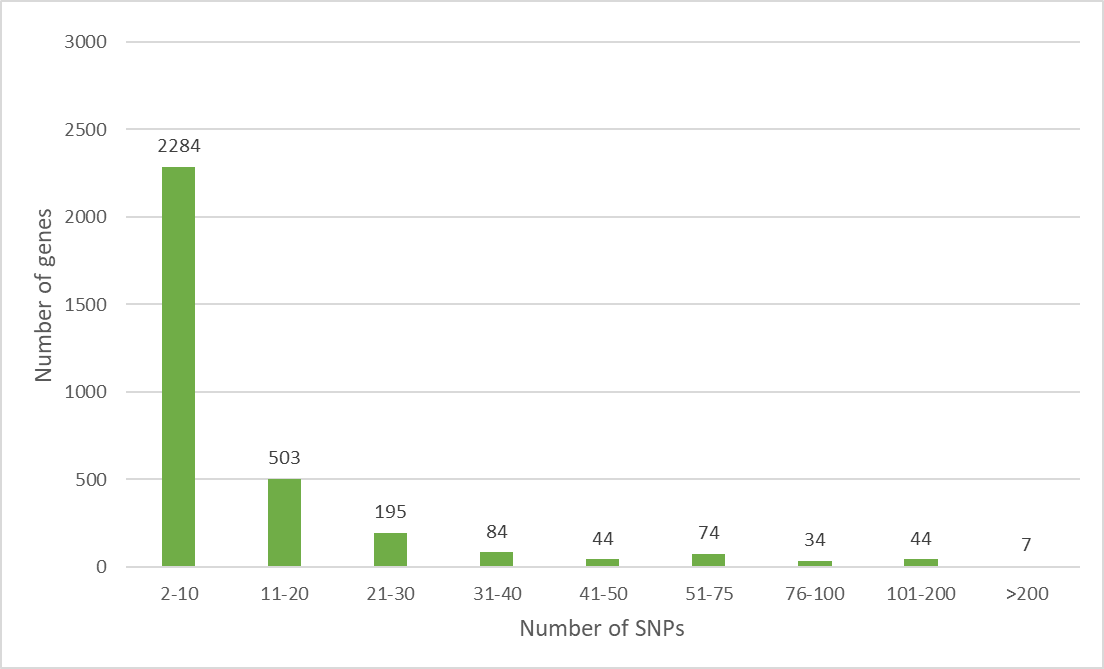
**Fig. S1** Summary for number of SNPs in each gene excluding genes with one SNP

**Fig. S2** Summary of significant results from GSA-SNP2, MAGMA and HisCoM-PCA for four phenotypes: T2D, HT, SBP and DBP


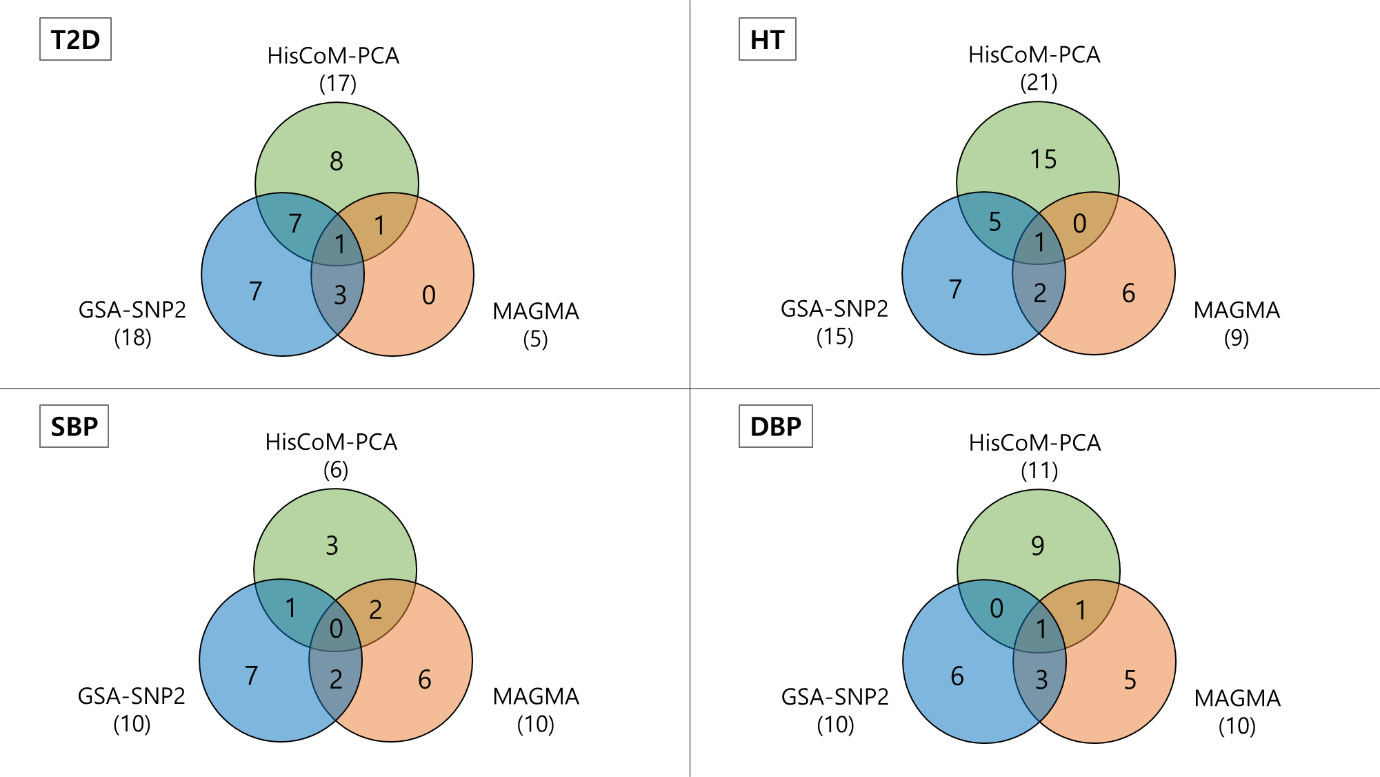


HisCoM-PCA includes two HisCoM-PCA results: one using the first PC and the other using the PCs whose cumulative proportion of variance is more than 30%. MAGMA is competitive version of MAGMA.
